# Hitting bacteria at the heart of the central dogma: sequence-specific inhibition

**DOI:** 10.1186/1475-2859-6-24

**Published:** 2007-08-10

**Authors:** Louise Carøe Vohlander Rasmussen, Hans Uffe Sperling-Petersen, Kim Kusk Mortensen

**Affiliations:** 1 Laboratory of BioDesign, Department of Molecular Biology, Aarhus University, Gustav Wieds Vej 10 C, DK-8000 Aarhus C, Denmark

## Abstract

An important objective in developing new drugs is the achievement of high specificity to maximize curing effect and minimize side-effects, and high specificity is an integral part of the antisense approach. The antisense techniques have been extensively developed from the application of simple long, regular antisense RNA (asRNA) molecules to highly modified versions conferring resistance to nucleases, stability of hybrid formation and other beneficial characteristics, though still preserving the specificity of the original nucleic acids. These new and improved second- and third-generation antisense molecules have shown promising results. The first antisense drug has been approved and more are in clinical trials. However, these antisense drugs are mainly designed for the treatment of different human cancers and other human diseases. Applying antisense gene silencing and exploiting RNA interference (RNAi) are highly developed approaches in many eukaryotic systems. But in bacteria RNAi is absent, and gene silencing by antisense compounds is not nearly as well developed, despite its great potential and the intriguing possibility of applying antisense molecules in the fight against multiresistant bacteria. Recent breakthrough and current status on the development of antisense gene silencing in bacteria including especially phosphorothioate oligonucleotides (PS-ODNs), peptide nucleic acids (PNAs) and phosphorodiamidate morpholino oligomers (PMOs) will be presented in this review.

## 1. Background

The antisense RNA (asRNA) mechanism comprises all forms of sequence-specific mRNA recognition leading to reduced or altered expression of a certain transcript [1]. Naturally occurring asRNAs are found in all three kingdoms of life, although most examples are found in bacteria, and they affect messenger RNA (mRNA) destruction, repression and activation as well as RNA processing and transcription [2]. This mechanism can be exploited in engineering strategies for inhibiting protein synthesis.

For the asRNA as well as other antisense compounds to be able to anneal to an mRNA or functional RNA, such as ribosomal RNA, the chosen RNA target region must be accessible. Determining target accessibility is the first step in designing antisense molecules, and both experimental and computational approaches have been applied. A brief and inexhaustive presentation will be given here.

**Figure 1:**
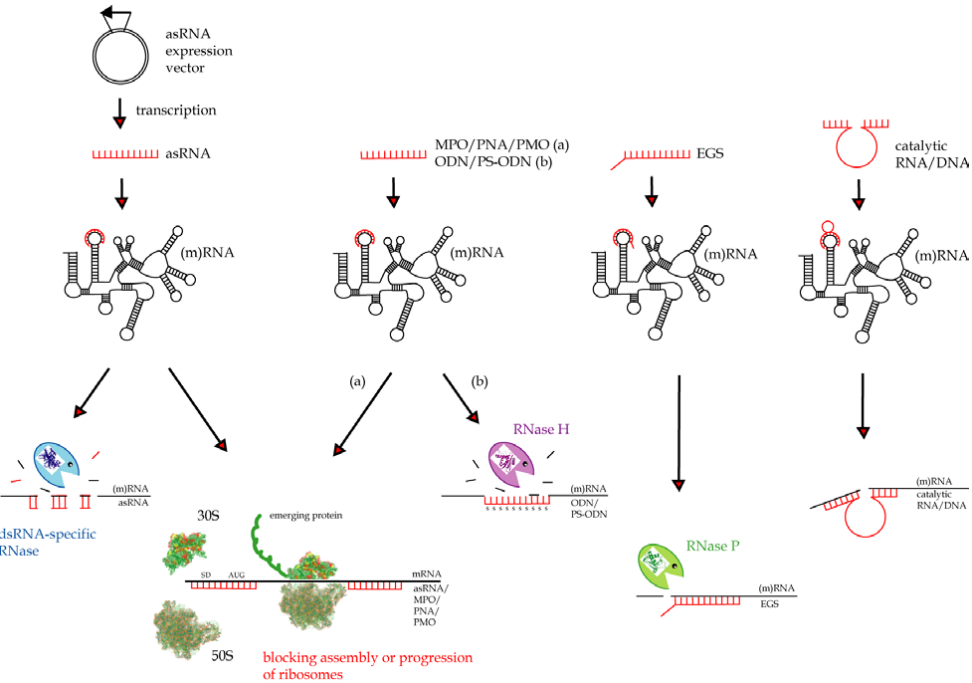
**Antisense inhibition mechanisms**. An overview of the antisense inhibition mechanisms described in this review. AsRNA inhibition mechanism is thought to include steric hindrance of mRNA translation or rapid degradation of target RNA possibly by dsRNA-specific RNases. MPOs, PNAs and PMOs also inhibit expression by sterically blocking translation. ODNs and PS-ODNs induce cleavage of target RNA by RNase H, while EGSs guide RNase P in target RNA cleavage. Catalytic RNA or DNA cleaves the target RNA upon hybridization. See the text for details.

When antisense molecules anneal to a complementary mRNA, translation can be disrupted as a result of steric hindrance of either ribosome access or ribosomal read-through (Fig. 1). This inhibition mechanism is, of course, specific for mRNAs. The annealing of antisense molecules to either mRNAs or functional RNAs can result in fast degradation of duplex RNA, hybrid RNA/DNA duplex, or duplex RNA resembling precursor tRNA by ribonucleases in the cell, or by cleavage of the target RNA by the antisense compound itself. The inhibitory efficiency of these hybridization strategies depends on factors such as length and structure, binding rate, intracellular concentration, and degradation resistance of the chosen antisense molecule. Antisense molecules and methods have been developed and designed specifically to address these issues and improve the inhibitory efficiency. These antisense strategies will be presented as well as recent studies describing their application.

Although the basic principle of the antisense inhibition mechanism is the same for all three kingdoms, the conditions affecting the efficiency of the particular antisense molecule are different in each system. Antisense gene silencing in bacterial systems will be the focus of this review. Application of antisense inhibition methods in eukaryotic systems, including siRNA and the RNA interference mechanism, is reviewed elsewhere [3-7]. An RNA-interference-based immune system in prokaryotes has been proposed recently [8,9], but a technology exploiting this finding for the sequence-specific inhibition of bacterial proliferation has still to be developed.

## 2. The target

It is not possible to design antisense molecules without investigating the RNA target. The target is an unchangeable RNA molecule with a defined structure, which must be examined in order to detect appropriate local target sites suited for invasion of an antisense strand [10]. Consequently, it is possible that the choice of target RNA limits the degree of inhibition obtainable [11].

The only method that will give you the precise answer, as to which target region is best suited for antisense inhibition, is empirical screening. However, this may be a tedious task, and ways of limiting the search area have been suggested.

Different experimental procedures have been applied to determine accessible mRNA regions, including chemical modification mapping [12] and *in vitro *screening assays using antisense oligodeoxyribonucleotides (ODNs). The ODN-mRNA duplexes are subjected to RNase H cleavage followed by gel electrophoresis [13,14], primer extension [15] or MALDI-TOF mass spectrometry [16] to identify cleavage sites. ODN inhibition efficiency can also be tested in *in vitro *transcription/translation experiments, in which the amount of expressed gene product is quantified following incubation with ODN [14,17].

**Figure 2:**
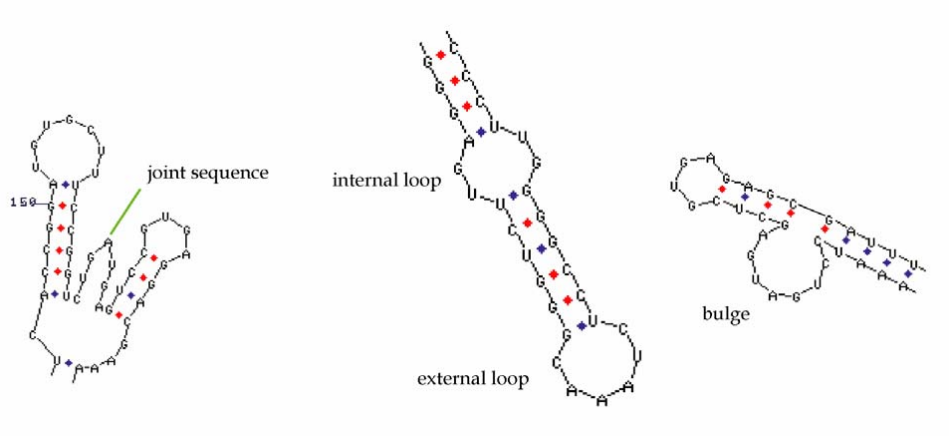
**Secondary structures favorable for invasion of antisense molecules**. Accessible regions include internal and external loops, bulges and joint sequences (secondary structures by Mfold).

RNA secondary structure predictions can be carried out by computer-assisted free energy minimization programs (like Mfold [18]) or structure sampling algorithms (like Sfold [19]) (for reviews see [20-22]) to identify accessible regions (Fig. 2). However, secondary structure predictions as well as *in vitro *experiments may be inaccurate due to unpredictable RNA-protein interactions that may change the RNA structure in the cells, as well as the coupling of transcription and translation rendering part of the mRNA absent for secondary structure formation. Scherr and co-workers developed a system for carrying out ODN-RNA binding experiments and endogeneous RNase H-mediated cleavage in cell extracts resembling the environment of the intact cell [23-26]. This approach resulted in ODN design with cell extract efficiency correlating with *in vivo *efficiency.

Antisense molecules should be targeting the start codon and the Shine-Dalgarno sequence of an mRNA [27]. This is suggested, since i) this region is accessible for ribosome assembly, ii) it is the usual target of natural examples of antisense inhibition, iii) for attenuated mRNAs this region is sequestered within a double-stranded region [28] and iv) consistent success has been experienced targeting this region. Furthermore, an mRNA 'sequence-walk' using 90 synthetical antisense probes pointed to the start codon region as the most sensitive to inhibition [29].

As mentioned before, the target of antisense molecules need not be mRNA. ODNs have been used to determine suitable target sites in catalytic RNA, such as bacterial RNase P RNA, by directly testing the inhibitory effect of the ODNs on the *in vitro *cleavage efficiency of the RNase [30]. Accessibility maps have also been produced for 16S [31] and 23S rRNA [32] of *Escherichia coli *by the use of ODNs labelled with fluorescent dye.

The results obtained by other researchers are valuable information, which others may build upon. Effective and ineffective ODNs reported in the literature have been entered into a database, from which they can be retrieved using two web-based retrieval tools [33]. This provides information on accessible and inaccessible sites in the targeted RNAs. The database contains ~700 ODNs targeting 46 RNAs, but unfortunately only one of these is an *E. coli *RNA, viz. 23S rRNA. If the database is regularly updated with ODNs targeting prokaryotic RNAs, it can prove a valuable tool in the target selection process in the future.

## 3. Expressed regular antisense RNA

Regular antisense RNA refers to the most basic form of asRNA, i.e. an unmodified RNA molecule of the exact reverse and complement sequence of the target RNA. To obtain this, a segment of the target gene can be amplified and cloned into a vector in an antisense orientation downstream from an inducible promoter. Regular asRNA is then introduced to the bacteria through transcription from this asRNA-encoding vector. The half-life of a typical bacterial mRNA is in the range of 2–4 min [34], and since most artificial asRNAs are usually subjected to rapid degradation [35], transfection of bacterial cells with an appropriate antisense gene vector is a way of providing a long-term expression downregulation.

The inhibition mechanism is thought to include steric hindrance of translation or rapid degradation of target RNA possibly by RNases specific for double-stranded RNA (dsRNA) (Fig. 1). It has been shown in *Staphylococcus aureus *that when targeting an mRNA using asRNA, the 5' portion of mRNA is completely degraded, while the 3' portion remains intact [36]. This might be due to the terminator stem-loop protecting the 3' end from exonucleases.

Studies on asRNA length dependence have shown a positive correlation between the percentage of inhibition and the length of antisense/target RNA hybridization [35], i.e. the longer asRNA and the longer target hybridization, the greater inhibition. This has also been suggested based on calculations of association rate constants [37]. In *Streptococcus mutans*, expressed asRNA larger than approximately 5% of the entire gene length has been indicated to be required for optimal inhibition of gene expression [38]. However, this length dependence may be specific for the studied target sequences, since shorter asRNAs have been shown to have a larger effect on other target genes [39]. Short asRNAs may suffer from ribosome displacement, since the ribosome is used to encounter mRNA secondary structures during translation. It has recently been shown that the ribosome exhibits helicase activity [40]. Downstream secondary structure resulting from annealed oligonucleotides of up to 27 bp could be disrupted by the ribosomal helicase activity, but this helicase activity appears not to function during translation initiation [40]. Consequently, asRNA annealing to the ribosome binding site (RBS) region should have a larger inhibitory effect than asRNA annealing further downstream on the mRNA.

The endogeneous expression of vector-induced asRNA-encoding genes is complicated by stretches of flanking vector-derived sequences, which are transcribed with the asRNA, why a low ratio of vector-derived sequences to complementary sequences is recommended [41]. This can be achieved by applying an appropriate cloning strategy or by introducing self-cleaving elements into the RNA transcript (see section 4).

Since long RNA molecules may exhibit more extensive secondary structure and may be more structurally complex, the observations of increased inhibitory effect when using longer asRNA molecules may imply that binding occurs between folded RNAs [11]. Studies on naturally occurring asRNAs have shown that complete pairing of an antisense and a target RNA is slow and can be unnecessary for inhibition [42]. Consequently, it may be contributive to include specific secondary structure elements in asRNA design, since unfolding of intrinsic secondary structure and complete duplex formation may be difficult and unnecessary. Naturally occurring asRNAs have provided researchers with model systems for asRNA structural design. For instance, different groups have designed asRNAs based on secondary structure elements found in CopA [11,43]. CopA, a naturally occurring asRNA, and its target, CopT, are key elements of the copy number control circuit of bacterial plasmid R1 [44-48], and a stem-loop structure resembling stem-loop II of CopA was incorporated into asRNA for rapid target recognition. However, most artificially created asRNAs are still about 100-fold less effective than natural asRNAs [11], signifying that natural asRNA regulation is not easily copied.

Computational strategies have been used extensively to search asRNA sequences for desirable features. AsRNA sequences can be selected out of the complete antisense sequence space, and a computer algorithm can be used to generate all possible asRNA sequences, usually within a specified range of sequence length. Secondary structure predictions are recorded (e.g. using the program Mfold [18]), and the lowest free energy foldings are selected. Different selection criteria for favorable antisense sequences have been suggested:

• a high number of terminal unpaired nucleotides [49]

• overall flexibility [49]

• a high number of external bases (i.e. nucleotides not involved in base pairing or structural elements) [41]

• a high number of structural components (i.e. structural folding units, regions of high complementarity within an RNA molecule) [41]

• a low ratio of the number of components to the number of total nucleotides [50].

The common denominator seems to be a high number of free nucleotides accessible for base pairing.

Effective asRNAs have been designed using computational strategies and algorithms, but the results may be affected by the specific algorithm used to calculate RNA secondary structure, the number of lowest energy structures that are considered, the length of target sequence segments, the selection criteria etc. [51].

Inhibition efficiency is also influenced by reaction kinetics, and since asRNA efficacy in living cells is related to annealing kinetics *in vitro *(shown for naturally occurring asRNA, [45,48]), kinetic selection techniques yielding fast annealing antisense species can be applied to generate effective asRNAs [52-54]. To further promote rapid recognition, a high ratio of asRNA to target RNA is desirable, and this can be accomplished by transfecting cells with antisense genes cloned into high copy number plasmids [35]. Furthermore, to prolong asRNA half-life and increase the intracellular concentration, 5' stem-loops can be inserted to protect RNAs from degradation [55-59].

### 3.1 Applications of expressed regular antisense RNA

The expressed asRNA strategy has mainly been used to study gene function. If the gene in question encodes an essential protein, knockout mutants will be lethal, whereas inducible and titrable downregulation of gene expression by asRNA encoded by a plasmid will enable functional studies. This strategy has been applied for the study of specific genes in a number of different bacteria including *E. coli *(*rpoS*) [60], *Mycobacterium smegmatis *(*hisD*) [61] and *Mycobacterium bovis *(*ahpC*) [62], *S. mutans *(*sgp*) [63], *S. aureus *(*srrAB*) [64], *Clostridium cellulolyticum *(*cel48F*) [65], *Thermus thermophilus *(*cat*) [66], *Lactobacilllus rhamnosus *(*welE*) [67], *Borrelia burgdorferi *(*ftsZ(Bbu)*) [68], *Helicobacter pylori *(*ahpC*) [69] and even in the protozoan parasite *Entamoeba histolytica *(*PIG-L*) [70]. These studies involved specific genes, but the asRNA expression strategy has also been used for genome-wide studies, in which the bacterial genome is randomly fragmented (~200 to 800 bp) and cloned into vectors. Screening transformants for conditional growth defective phenotypes and characterizing the corresponding expressed antisense fragments has led to the identification of essential genes in *S. aureus *[71-73] and *S. mutans *[38] and genes essential to both *S. mutans *and *E. coli *[74].

Changing the expression profile of bacteria can have other purposes. Metabolic engineering of bacteria can improve their use as fermenters and cell hosts for recombinant heterologous protein production. For instance, an increase in the butanol-to-acetone ratio in *Clostridium acetobutylicum *fermentations was obtained using expressed asRNA specific for proteins in the involved pathways [50,75,76]. In another study, asRNA was used to reduce synthesis of a glycosyltransferase, which resulted in a change in the molecular mass of the polysaccharides produced in *L. rhamnosus *[67]. In *E. coli*, different strategies have been explored in order to increase recombinant protein production yield, including downregulating the global regulator σ^32 ^to decrease proteolytic degradation of recombinant protein [77], downregulating RNase E to stabilize target product mRNA [78], and reducing endogeneous acetate production to improve heterologous protein synthesis [79] without concomitant growth inhibition. Acetate can cause inhibition of growth and recombinant protein synthesis, but the acetate pathway is also physiologically indispensable, which is why asRNA downregulation of enzymes of the acetate pathway is preferred over gene knockout. Additionally, asRNA expression can be used to protect bacteria used in industrial fermentations against bacteriophages (for reviews see [80-82]).

The expression of asRNA has also been used to validate the point of action of antibiotics. Targeting the proposed molecular target of an antibiotic by asRNA and reducing its expression will sensitize the bacteria to this specific antibiotic. This strategy has especially been used in *S. aureus *to validate mode of action of antibiotics [72,83] as well as to screen for novel antibacterial agents [36,84-89], but it can also be used to restore antibiotic susceptibility of other resistant strains [90].

Alpha-toxin of *S. aureus *is well-established as a lethal toxin in mice [91]. A 14- and 16-fold reduction in alpha-toxin expression has been achieved in *S. aureus *cell cultures by introducing asRNA against the gene encoding this toxin [92,93]. The effect has also been tested in murine models. Mice were infected with the transformed *S. aureus *strains, and inducer was administered orally [92]. The asRNA expression was shown to eliminate the lethality of the infection. This lead to the proposal of using this strategy to create live-attenuated strains for vaccine candidates [93].

In all the studies mentioned so far, the expressed asRNAs have been complementary to their target mRNAs in an antiparallel orientation. However, expression of parallel asRNA targeting the *E. coli lon *mRNA has been shown to interfere with target protein synthesis [94] but has not been further investigated.

## 4. External Guide Sequences

**Figure 3:**
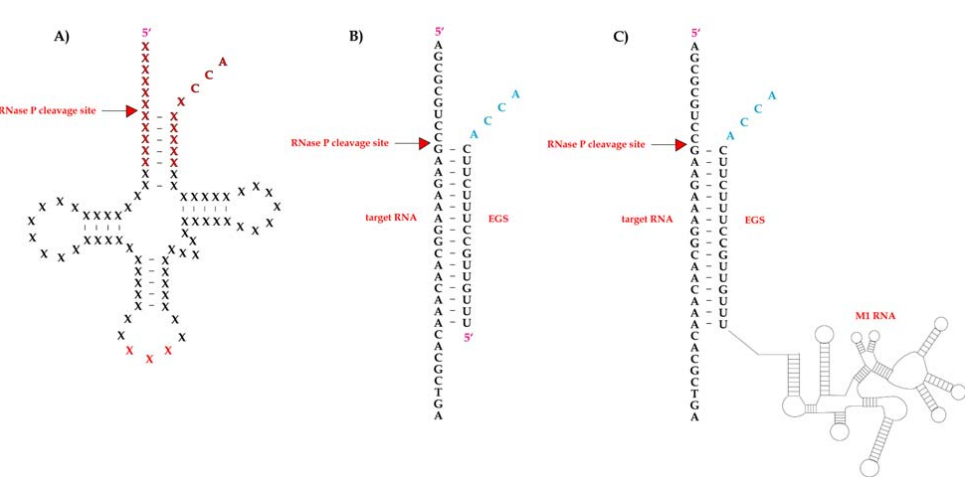
**External Guide Sequences**. A) Representation of precursor tRNA, a natural substrate for *E. coli *RNase P. The anticodon is shown in red, and the sequences imitated by EGS annealing are highlighted in boldface. B) An EGS molecule annealing to an RNA substrate. C) An EGS molecule with an attached catalytical M1 RNA subunit annealing to an RNA substrate. Arrows mark the RNase P or M1 RNA cleavage sites.

External guide sequences (EGS) are small asRNA molecules, which hybridize to their target RNA to create a structure that mimics a naturally occurring cleavage substrate of RNase P (Fig. 1). RNase P is an essential enzyme, which is found in all organisms, and it is also one of the most abundant and active enzymes [95]. In *E. coli*, this ribonucleoprotein consists of a catalytic M1 RNA subunit [96] and a C5 protein subunit, which is a necessary cofactor *in vivo *[97]. RNase P is responsible for generating mature 5'ends of tRNAs by a single endonucleolytic cleavage of their precursors [98], and this cleavage is not sequence-specific but depends on a higher order structure in precursor tRNA (ptRNA) [99]. RNase P cleavage of one of the strands in a bimolecular substrate resembling ptRNA structure has been demonstrated [100]. Actually, the model substrate could be simplified to consist of two complementary RNA strands forming a stem-like structure of typically 13–16 bp [100,101], and this led to the development of EGSs (Fig. 3).

Since the target mRNA will be one of the two strands in the model substrate, the EGS is designed to serve as the complementary RNA strand, guiding the RNase P cleavage.

Some criteria must be met by the target mRNA and the EGS to ensure sufficient ptRNA mimicry: i) the 3'end of the EGS must contain an unpaired RCCA sequence [102] (and consequently, positions -2 and -3 of the mRNA cleavage site must not be guanosines), ii) the mRNA must contain a guanosine 3' to the cleavage site and iii) a pyrimidine 5' to the cleavage site [103,104]. Furthermore, if the 3' RCCA sequence is followed by a long flanking region, RNase P cleavage efficiency is markedly reduced [105], and this will often be a problem when expressing EGSs from a transfected plasmid.

**Figure 4:**
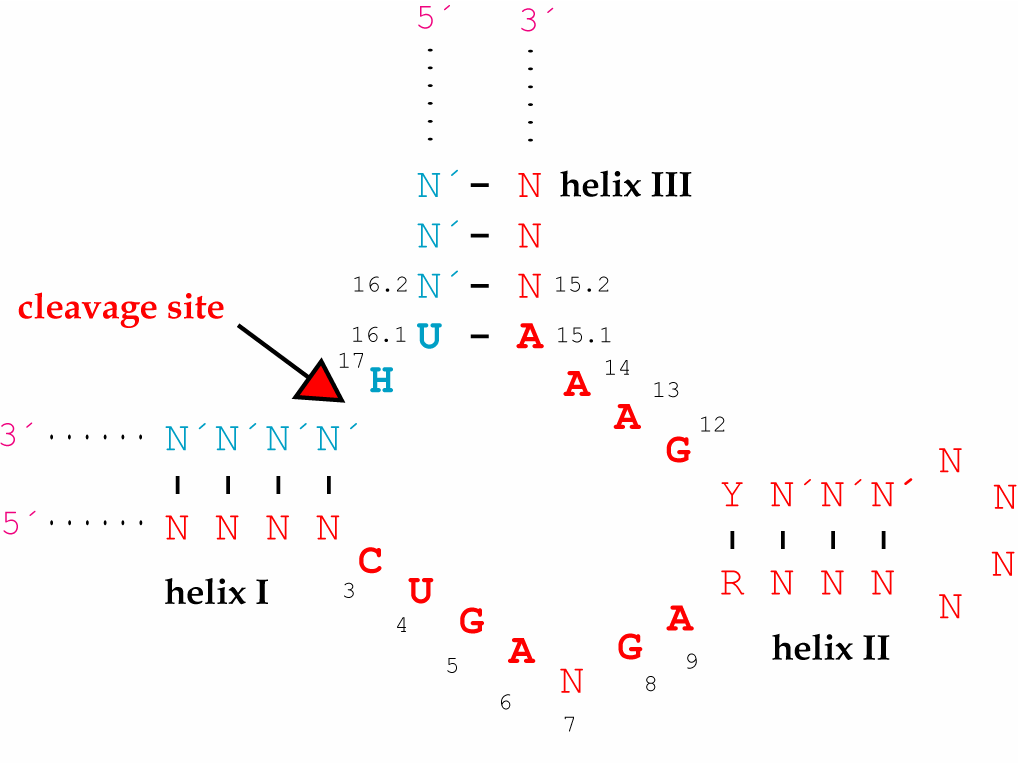
**Hammerhead core sequence**. Generalized representation of a hammerhead-substrate complex. N: any nucleotide, N': nucleotide complementary to N, H: any nucleotide but G, Y: pyrimidine nucleotide, R: purine nucleotide complementary to Y. Nucleotides in boldface indicate the conserved catalytic core nucleotides, and the arrow marks the cleavage site. Ribozyme numbering is according to Hertel *et al*. [219]. This representation displays *in trans *cleavage, but the catalytic core nucleotides and the cleavage site are the same for *in cis *cleavage.

**Figure 5:**
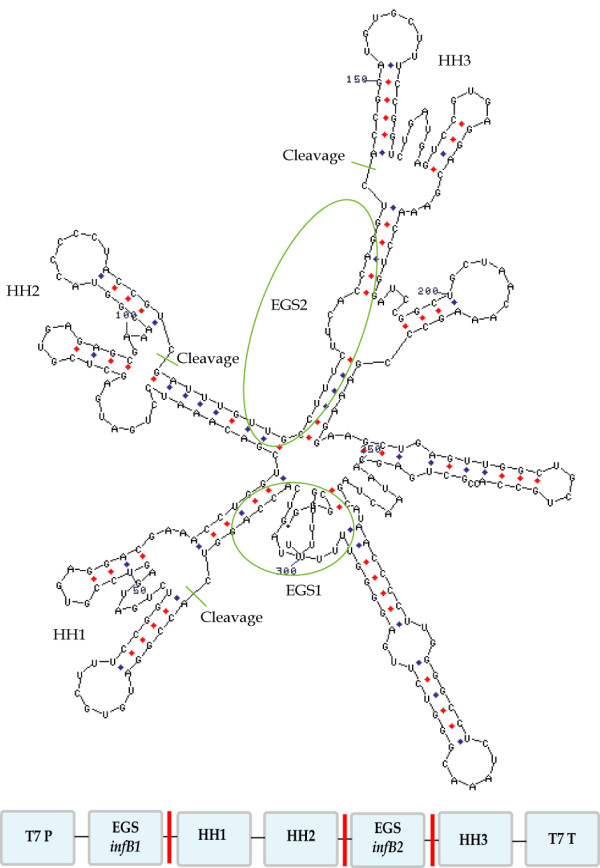
**Using hammerhead sequences to release EGSs**. Mfold structure prediction of the plasmid-encoded sequence EGS1-HH1-HH2-EGS2-HH3-T7 terminator constructed for inhibition of IF2 synthesis in *E. coli *(LCV Rasmussen, unpublished results).

To circumvent this problem, the EGS-encoding sequence can be followed by a hammerhead-encoding sequence (HH sequence) [106,107]. When this long transcript is produced, the hammerhead sequence will form a specific secondary structure and self-cleave, generating a free EGS molecule with only few nucleotides 3' to the RCCA sequence [105]. The general structure of a hammerhead sequence is illustrated in Fig. 4, in which the conserved core nucleotides needed for efficient cleavage are highlighted in boldface. The hammerhead sequences are also useful when expressing two EGSs from the same plasmid, in which case two hammerhead sequences of opposite direction are required between the two EGSs and a third one downstream from the last EGS, as illustrated in Fig. 5.

### 4.1 Applications of External Guide Sequences

EGS inhibition of protein synthesis was first investigated in an *E. coli *plasmid system [99,105]. Alkaline phosphatase and β-galactosidase mRNAs were targeted using single EGSs followed by hammerhead sequences and EGSs covalently linked to M1 RNA. The M1 RNA-EGSs cleaved target mRNA independently of RNase P.

EGS expression has been used for phenotypic conversion of antibiotic-resistant bacteria. Expressing EGSs targeting the resistance-conferring genes of chloramphenicol acetyltransferase (*cat*), β-lactamase (*bla*) [99,108] and aminoglycoside 6'-*N*-acetyltransferase type Ib (*aac(6')Ib*) [109] restored antibiotic sensitivity, and it was demonstrated that a high EGS-to-target mRNA ratio is required for efficiency [108,110].

Bacterial growth can also be inhibited by targeting essential genes, as e.g. the genes encoding C5 protein subunit of RNase P (*rnpA*) and subunit A of gyrase (*gyrA*) in *E. coli*. Multiple EGSs targeting these to mRNAs showed additive effects and resulted in a 26-fold decrease in growth after EGS induction, the highest growth inhibition achieved in this study in wild-type *E. coli *[110].

The results obtained in *E. coli *has been transferred to *Salmonella enterica *serovar Typhimurium [111]. The non-essential target genes *invB *and *invC *express proteins required for type III secretion and for host cell invasion, respectively. EGS targeting of these genes resulted in a decreased host cell invasion rate.

Since the EGSs are expressed from plasmids, the bacteria may try to lose these plasmids to avoid inhibition [109]. Plasmid loss in liquid cultures of bacteria have been shown to begin after 6 hours of growth [108]. In another study transformants containing EGSs with high inhibition index values lost their plasmids more often, exhibited smaller colony size when induced and were more difficult to grow in general than the corresponding low inhibition index EGS transformants [110]. It would seem that the bacteria, when exposed to more effective EGSs, will try harder to escape the growth-inhibiting agents. For *in vitro *applications, the use of antibiotic resistance markers is routinely used to prevent loss of plasmid, but the EGSs may also be administered directly as chemically synthesized molecules, avoiding the problem of plasmid loss.

Even though the results described here are obtained through induced EGS expression from plasmids transformed into bacteria, the technology can, in principle, be transferred to *in vivo *applications. Guerrier-Takada *et al*. suggest altering the so-called R-plasmids of enteric bacteria and derivatives, which are responsible for establishment and transfer of drug resistance in clinical populations, to carry EGS-encoding genes instead of genes for drug resistance [108]. Another option may be to use carriers such as fluid liposomes, which has been shown to facilitate *E. coli *cell entry of both plasmid DNA and antisense oligonucleotides [112]. Chemically synthesized EGSs could be added instead of plasmid DNA, and the EGSs could be DNA-based instead of RNA-based to increase nuclease stability. However, DNA-based EGSs have been shown to be 10-fold less efficient *in vitro *than unmodified RNA-based EGSs [113].

Using EGS technology for microbial antisense inhibition still needs further development and optimization, and some of the modifications investigated in human cells are still to be investigated in bacterial cells. It should be kept in mind, though, that due to differences in the structural requirements of EGSs directing mRNA cleavage by either human or bacterial RNase P, the results obtained in one system may not be transferred to the other [99].

## 5. Catalytic antisense and its applications

The hammerhead self-cleaving domain, which was just briefly mentioned above, was first discovered in plant viroids [114-116], and it was soon demonstrated that this structure could be dissolved into two strands, one of them directing the *in trans *cleavage of the other [117,118]. As shown in Fig. 4, *trans*-acting hammerhead ribozymes form helices I and III with complementary sequences in the substrate, whereas helix II is an intramolecular structure of the ribozyme. Truncation tests have revealed that helix length can be varied for optimization of cleavage efficiency [119-121].

The use of catalytic antisense RNA in bacterial systems has been limited. The explanation for this might involve the tight coupling of transcription and translation in prokaryotes. A plasmid-encoded hammerhead ribozyme targeting chloramphenicol acetyltransferase (*cat*) gene was only efficient in mutant strains of *E. coli *[122], in which the translation rate was decreased so as to uncouple translation from transcription.

The self-cleaving hammerhead structure has instead been used indirectly in bacterial growth inhibition, by releasing EGSs from long RNA transcripts as described in the previous section.

**Figure 6:**
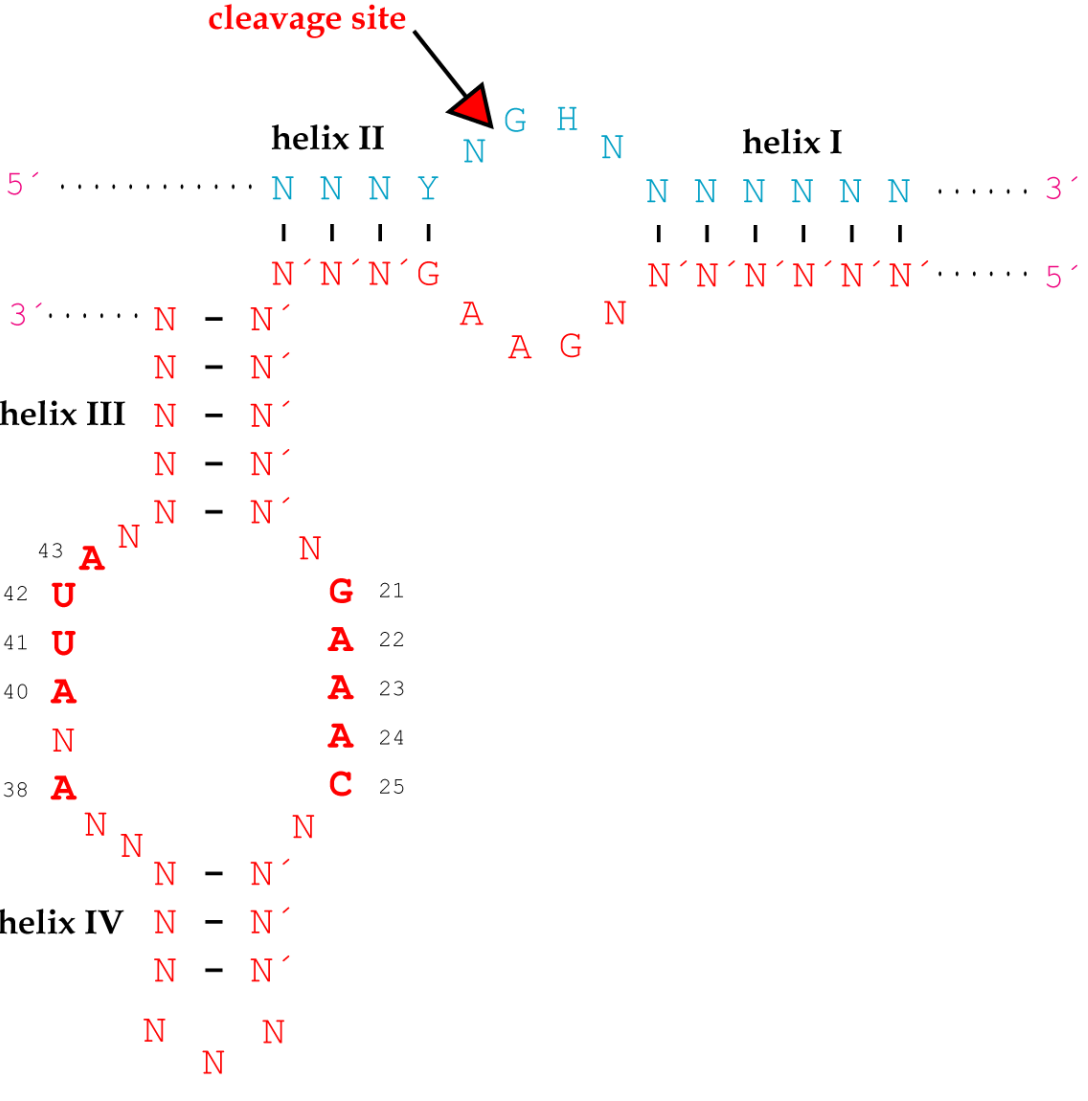
**Hairpin core sequence**. Generalized representation of a minimal hairpin-substrate complex. N: any nucleotide, N': nucleotide complementary to N, H: any nucleotide but C, Y: pyrimidine nucleotide. Nucleotides in boldface indicate the conserved catalytic core nucleotides, and the arrow marks the cleavage site. Ribozyme numbering is according to Butcher and Burke [220, 221]. This representation displays *in trans *cleavage, but the catalytic core nucleotides and the cleavage site are the same for *in cis *cleavage.

The hairpin motif is a somewhat larger catalytic RNA motif, and it was first discovered in the negative strand of the tobacco ringspot virus satellite RNA, (-)sTRSV [123-125]. As for the hammerhead motif, the hairpin motif could be dissolved into two separate strands, one of them directing the *in trans *cleavage of the other. The secondary structure of the minimal hairpin ribozyme involves two arms hybridising to the target sequence in addition to two intramolecular helices, as shown in Fig. 6 (for further details on hairpin ribozyme structure see [126-128] and references herein).

The hairpin ribozyme has not been a common object of studies in prokaryotes. Hairpin ribozymes produced in *E. coli *showed only a minor or negligible effect [11]. This might be a result of the chosen target for the ribozymes, since ribozymes targeting downstream from the RBS region might be displaced by the translating ribosome.

**Figure 7:**
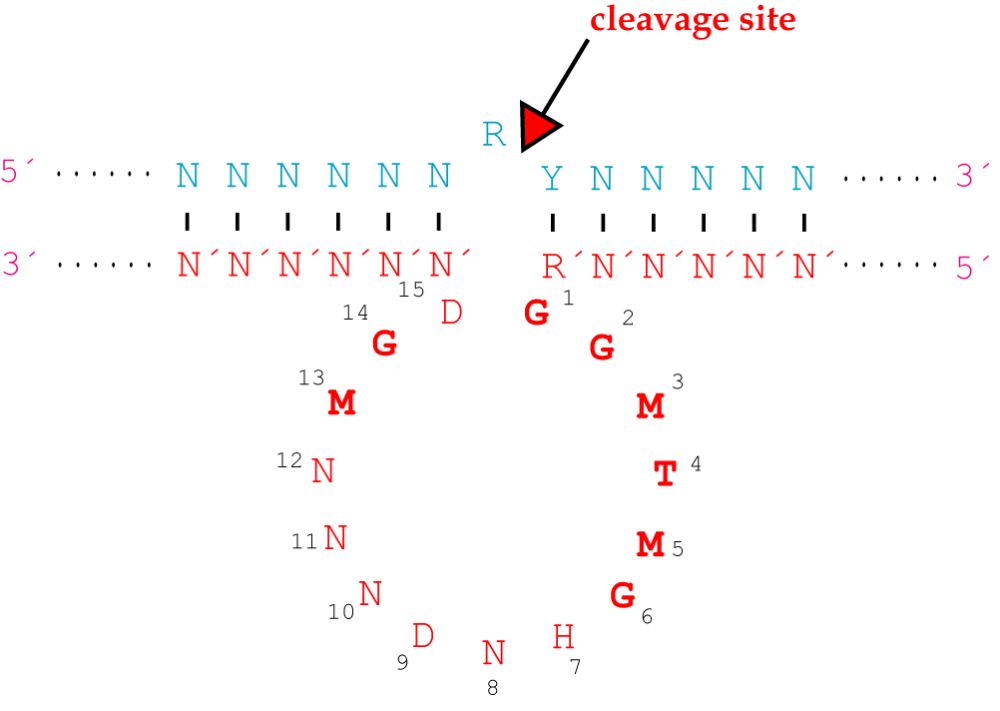
**10–23 DNA enzyme**. Generalized representation of a 10–23 DNA enzyme-substrate complex. N: any nucleotide, N': nucleotide complementary to N, R: purine nucleotide, Y: pyrimidine nucleotide, R': purine complementary to Y, M: A or C, H: A, C or T and D: G, A, or T. The nucleotides likely to be directly involved in forming the catalytic site are highlighted in bold, and the arrow marks the cleavage site. Numbering is according to Zaborowska *et al*. [222].

RNA ribozymes may be subjected to rapid degradation by nucleases, so catalytic DNA has also been investigated. The most prominent deoxyribozyme is the so-called 10–23 DNA enzyme (Fig. 7), which was obtained by *in vitro *selection from a combinatorial library [129]. It is composed of about 30 deoxynucleotides, 15 in a catalytic core region and 6–12 in each of two flanking arms complementary to the target RNA. It catalyzes an Mg^2+^-dependent cleavage between an unpaired purine and a paired pyrimidine [130]. Deletion analysis showed that the bases at positions 7 and 8 could be deleted without severely impacting the catalytic activity of the enzyme [131].

**Figure 8:**
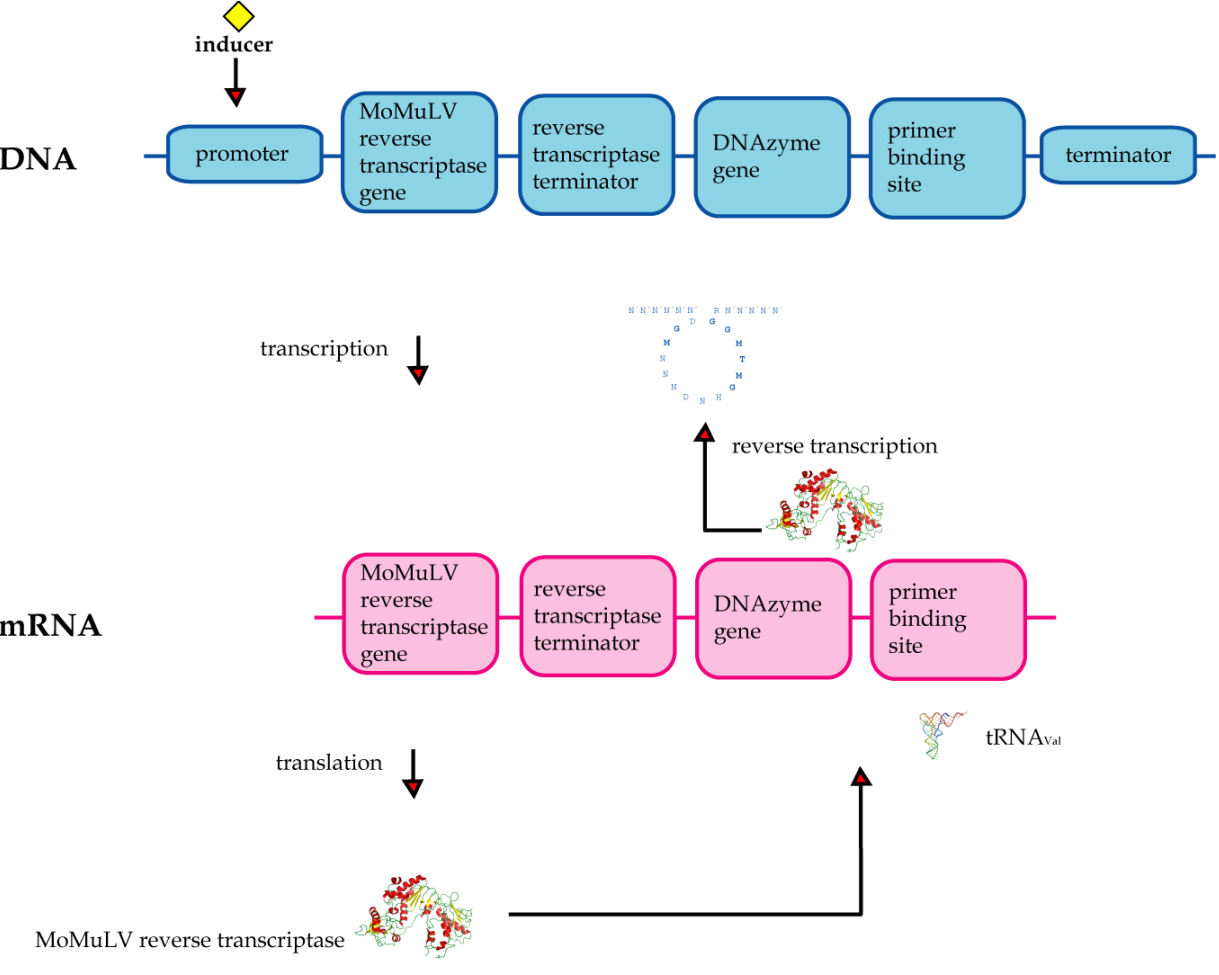
**Synthesis of single-stranded DNAzymes**. The ssDNA was synthesized by a reverse transcriptase from moloney murine leukemia virus (MoMuLV), the gene of which was placed downstream from a hybrid P_LtetO-1 _promoter in the vector. This was followed by coding sequences for a reverse transcription termination structure, a DNAzyme, a primer binding site (PBS), and a terminator. The MoMuLV reverse transcriptase was synthesized from the resulting transcript encoding reverse transcriptase, reverse transcription terminator, DNAzyme, and PBS. Endogeneous tRNA(Val) then hybridized to the PBS and primed the reverse transcription of the single-stranded DNAzyme, which was stopped by the reverse transcription terminator.

Chen *et al*. developed a vector system for the *in vivo *expression of single-stranded DNA (ssDNA) and DNA enzymes (DNAzymes) in mammalian cells [132-134], which was recently transferred to *E. coli *[135]. Using this system, ssDNA was synthesized by reverse transcription from a specific vector (see Fig. 8 for details), and the encoded DNAzyme was a 10–23 DNAzyme targeting the *ftsZ *gene essential for bacterial division and viability. *FtsZ *expression level in *E. coli *cells was reduced significantly upon induction, and cell growth was inhibited in a time- and inducer concentration-dependent manner [135].

Ribonucleases can also be produced synthetically. Artificial ribonucleases composed of building blocks of different features have been developed and tested for *in vitro *cleavage of RNA [136,137].

## 6. Antisense oligonucleotides, analogues and mimics

**Figure 9:**
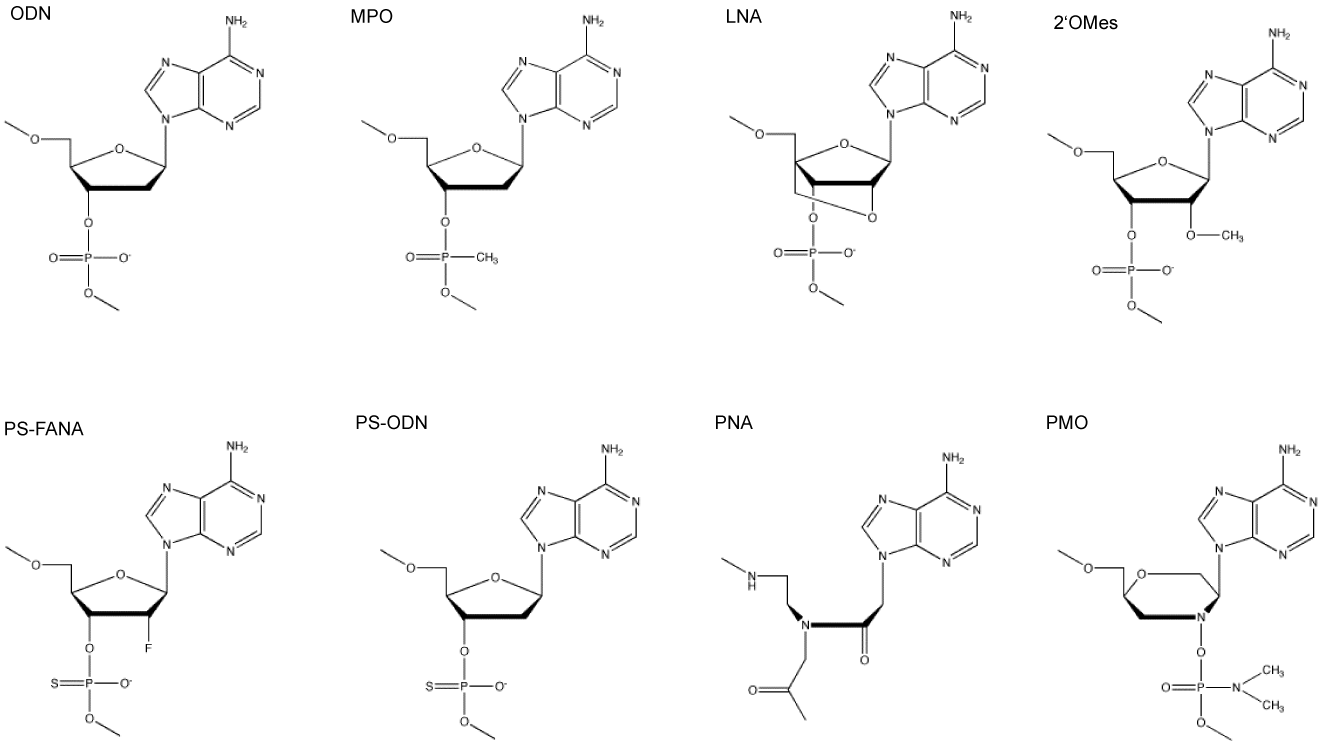
**Structures of antisense building blocks**. Structures of the building blocks for unmodified oligodeoxynucleotides (ODNs), methylphosphonate oligonucleotides (MPOs), locked nucleic acids (LNAs), 2'-*O*-methylribonucleotides (2'-*O*Mes), phosphorothioate-linked 2'-deoxy-2'-fluoro-D-arabinonucleic acids (PS-FANAs), phosphorothioate oligonucleotides (PS-ODNs), peptide nucleic acids (PNAs), and phosphorodiamidate morpholino oligomers (PMOs). All examples include an adenine base.

Antisense phosphodiester oligodeoxyribonucleotides (asODN, Fig. 9) are short (usually 10–30 nucleotides) synthetic DNA sequences complementary to a given mRNA target. When hybridizing to mRNA, the heteroduplex is recognized by RNase H, and the RNA strand is degraded [138] (Fig. 1). This is the main mechanism of action, however, ODNs have also been used in an RNase H-independent strategy to bind and induce misfolding of catalytic RNA [139,140].

As described in section 2, the asODNs have mainly been used in cell-free or cell extract assays to determine accessible target sites in mRNAs as well as studying topology of ribosomal RNAs [141-144]. However, ODNs have been used in antimicrobial studies as well. ODNs have been shown to decrease the level of antibiotic resistance in *E. coli *by targeting the *aac(6')Ib *mRNA [14], which has also been targeted by EGS technology, as mentioned in section 4.

ODNs are subjected to rapid degradation by nucleases, so their use as antisense agents has been limited. Instead, synthetic DNA analogues with altered backbones have been developed to overcome some of the problems found with natural DNA oligonucleotides. The naturally occurring DNA bases are maintained in positions required for complementary base pairing, but the linkages between the bases have been modified to prevent degradation by nucleases, to improve cellular uptake, and increase binding affinity. Different linkages of these analogues have different advantages and effects, which will be reviewed here as well as the development of these DNA analogues as antimicrobial agents.

Methylphosphonate oligonucleotides (MPOs, Fig. 9) are formed by replacing one of the non-bridging oxygens of ODN phosphodiester bonds with a methyl-group. Besides the formation of noncharged oligonucleotides this also generates chirality at the phosphorus centres. Thus, MPOs can consist of phosphate backbones, in which either all phosphorus centres are in the R configuration (all-Rp, the p denominating the phosphorus centre) or S configuration (all-Sp) or a mixture. The all-Rp diastereomers have been indicated to form more stable duplexes than the all-Sp diastereomers or racemic MPOs [145-147].

MPOs act by an RNase H-independent inhibition mechanism (Fig. 1), and though highly stable, the reduced solubility and cellular uptake of methylphosphonate ODNs [138,148] have limited their use as antisense agents in bacteria [149,150].

A wide variety of DNA (or RNA) analogues (Fig. 9) have been designed and used in bacterial systems, e.g.

• methylcarbamate ODNs [151],

• photoactivatable ODNs [152],

• locked nucleic acids (LNA) [153,154],

• mixed-backbone ODNs composed of

- segments of phosphorothioate ODNs (PS-ODNs, see section 6.1) and 2'-*O*-methylribonucleotides (2'-OMe) [155] or

 - phosphorothioate-linked 2'-deoxy-2'-fluoro-D-arabinonucleic acid (PS-FANA) [156],

but also fundamentally different strategies have been investigated, e.g.

• oligonucleotide-directed misfolding of RNA [139],

• triplex-forming antigene ODNs [157,158] and

• expression of multicopy, ssDNA from retrons [159,160].

However, the DNA analogues and mimics that have been most widely used in bacteria and show the most promising results are the phosphorothioate ODNs (PS-ODNs), the peptide nucleic acids (PNAs) and the phosphorodiamidate morpholino oligomers (PMOs) [136,161,162], which are discussed in the following subsections.

### 6.1 Phosphorothioate oligonucleotides

Replacing one of the non-bridging oxygens of the phosphodiester bonds of DNA with sulfur gives phosphorothioate oligodeoxynucleotides (PS-ODNs, Fig. 9), which are some of the most studied oligonucleotides. As for the MPOs, the introduction of sulfur atoms introduces chirality at the phosphorus centers, and the PS-ODNs behave differently depending on the diastereomeric configuration of the linkages. Thus, the all-Rp version has a higher binding affinity and a greater RNase H activation but a lower *in vitro *stability against nucleases than the all-Sp diastereomer. PS-ODNs of mixed diastereomeric linkages show intermediate abilities [163,164]. It has been indicated that nuclease stability is one of the most important factors for PS-ODN efficacy [165], and hence the stereocontrolled synthesis of antisense PS-ODNs has been well investigated (for a review see [163]).

The PS-ODNs are highly soluble, and their mechanism of action involves binding to target RNA and activating RNase H for cleavage of the target (Fig. 1). RNase H-dependent ODNs can be targeted to virtually any region of the RNA or mRNA, whereas antisense agents inhibiting expression through steric blocking should be targeted to the 5' end or the initiation codon region [138]. The phosphorothioate DNA analogues can display poor sequence specificity, since even short duplexes of PS-ODN/RNA can be degraded by RNase H, and PS-ODNs can therefore induce cleavage of sequences with only partial homology to the targeted RNA [166]. They have also been shown to induce sequence-independent effects by interacting with cellular proteins, though this is suggested to be synergistic with the downregulating effect of the PS-ODN [138].

### 6.2 Applications of phosphorothioate oligoculeotides

**Figure 10:**
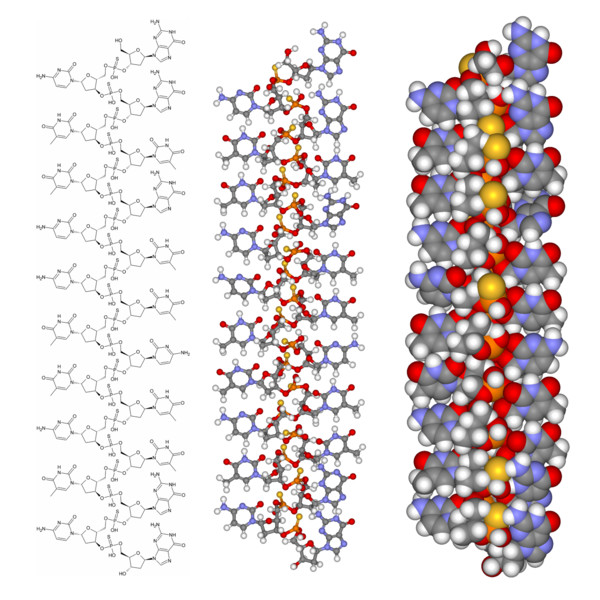
**The first FDA-approved antisense drug**. Structure of the 21-mer phosphorothioate, fomivirsen (brand name Vitravene, illustration from [223]). The patient target group for this drug is rather small, and it was taken off the market by the manufacturer in 2002 due to poor sales [224].

PS-ODNs have been studied and used extensively in eukaryotic systems, and this has led to the development of the first FDA-approved antisense drug, fomivirsen [167] (commercially known as Vitravene). The 21-mer PS-ODN (Fig. 10) designated ISIS-2922 targets the major immediate-early gene of human cytomegalovirus in HIV-patients with retinitis. More PS-ODN drugs for treatment of human diseases are in clinical trials, but the use of PS-ODNs in bacteria has been rather limited and nearly confined to *Mycobacteria *(Table 1).

**Table 1:** Studies on PS-ODNs in bacteria.

PS-ODN	Target	Test system	Reference
21–36-mer PS-ODNs	*ask*	*M. smegmatis *wt and drug-resistant in liquid culture	[180]
15–21-mer PS-ODNs	*marA *+ *marO*	*E. coli *liquid culture	[175]
18–24-mer PS-ODNs	*glnA1*	*M. tuberculosis + M. smegmatis *liquid culture	[168]
24–29-mer PS-ODNs	*fbpABC *+ *glnA1 *+ *alr*	*M. tuberculosis *liquid culture	[169]
18–19-mer PS-ODNs	*gtfB*	*S. mutans *liquid culture	[176]
21-mer PS-ODN	*ino1*	*M. tuberculosis *liquid culture	[173]

Applying PS-ODN technology to *M. tuberculosis *demonstrated several important parameters:

• concentrations of ≥10 μM were most effective (much higher concentrations are undesirable due to increase in non-sequence-specific interactions with host proteins and nucleic acids [168]),

• PS-ODNs should be at least 18 and preferably 24 bases in length to minimize random hybridization,

• and mRNA targets should have a low propensity to form stable secondary structure at 37°C (based on a secondary structure analysis program) [168,169].

This was elucidated from the targeting of three different sites in *gln*A1 mRNA encoding glutamine synthetase, the export of which is associated with pathogenicity and formation of cell wall structure [170]. Besides *gln*A1 [168], PS-ODNs have been used to target

• *fbpA*, -*B *and -*C *[169,171] (single operon genes encoding three proteins of the 30/32-kDa protein complex that are essential for mycobacterial cell wall synthesis and leading tuberculosis vaccine candidates [172]),

• *alr *[169] (encoding alanine racemase essential for synthesis of cell wall peptidoglycan)

• and *ino1 *[173] (encoding inositol-1-phosphate synthase, a key enzyme in phosphatidyl-inositol synthesis).

Combinations of PS-ODNs targeting these mRNAs demonstrated growth inhibition of *M. tuberculosis *and sensitized the cells to conventional drugs. Furthermore, it was most importantly demonstrated that bacterial growth could be inhibited in human macrophages infected with *M. tuberculosis*, albeit with a fairly low efficiency [171].

PS-ODNs have been used in a few other bacterial species. The *MecR1-mecI *gene of *S. aureus *regulates synthesis of penicillin-binding protein 2a (PBP2a), which mediates resistance to methicillin and all β-lactam antibiotics. A PS-ODN targeting *MecR1 *mRNA restored antibiotic susceptibility of a methicillin-resistant *S. aureus *(MRSA) strain [174].

Another example of restoration of antibiotic susceptibility comes from mutant *E. coli *strains, in which a PS-ODN restored a norfloxacin sensitive phenotype, although with lower efficiency [175].

In *Streptococcus mutans*, *gtfB *encodes glycosyltransferase, which is responsible for synthesis of water-insoluble glucans facilitating adhesion of the organism to the surface of teeth. The GtfB synthesis and activity as well as biofilm formation of *S. mutans *was inhibited by an anti-*gtfB *PS-ODN, but bacterial growth was unaffected [176]. Nevertheless, this might be useful in caries prevention.

In this study, uptake of PS-ODN and hence inhibitory efficiency in this Gram-positive bacterium was improved by adding a transfection reagent of cationic polymers, though inhibition was observed with free PS-ODN as well [176].

Mycobacteria are neither truly Gram-negative nor -positive [177]. Their outer lipid bilayer is the thickest biological membrane hitherto known, and the exceptionally low permeability of this membrane renders mycobacteria resistant to many antibiotics [178]. The ability of lipophilic drugs to solubilize within the lipid portion of the outer wall layer leads to improved efficiency over hydrophilic drugs, which are prevented from traversing the cell envelope [179]. Li *et al*. claim that the PS-ODNs should be more easily taken up due to improved lipophilicity [173].

In *M. smegmatis*, targeting the aspartokinase (*ask*) gene using PS-ODNs was only effective in the presence of low levels of ethambutol, which increases permeability of the cell wall [180]. As mentioned above, PS-ODNs of different targets have been shown to enter mycobacterial cells unassisted and inhibit growth. However, improvement of uptake is still necessary to further improve the efficiency. Physical methods like heat shock and electroporation have been used as well as mutant strains with a permeable membrane [174,175], but these methods are not suited for targeting wildtype bacteria in tissue. Combining administration of PS-ODNs with conventional antibiotics increasing cell permeability seems fairly straightforward. Different as well as related strategies involve

• covalently attaching PS-ODNs to D-cycloserine (antibiotic thought to be taken up by D-alanine uptake system) or biotin [180,181],

• tethering PS-ODNs to amikacin-moiety (antibiotic targeting 30S ribosomal subunit),

• "softening" the cell wall using subinhibitory concentrations of antibiotics [168],

• incorporation of terminal 2'-*O*-methyl ribose residues (promotes stability and diminishes animal toxicity),

• 3' addition of a nonhybridizing poly-G tail (postulated to improve uptake in eukaryotes) [169] and

• encapsulation into fluid liposomes [112].

Most of these strategies resulted in only slightly enhanced efficacy if any, so the ultimate uptake-enhancing strategy is yet to be developed.

### 6.3 Peptide Nucleic Acids

Peptide nucleic acids (PNAs, Fig. 9) were developed by Nielsen *et al*. in 1991 [182]. They are nucleic acid analogues, which are based on an aminoethylglycin backbone with acetyl linkers to the nucleobases [183]. This very flexible pseudopeptide backbone is uncharged and thus avoids the electrostatic repulsion, a natural phosphate-ribose backbone would induce during hybridisation. This is thought to be the reason, why these PNAs can form very stable duplexes or triplexes with either single-stranded or double-stranded DNA or RNA [138]. Another consequence of this unnatural backbone is that the PNAs are not degraded by enzymes such as nucleases and proteases [184], and they are also not recognized by RNase H, so the mechanism of action involves binding and steric blocking of ribosomal assembly and translation (Fig. 1). However, being neutral and only slightly hydrophilic molecules, PNAs suffer from low aqueous solubility compared to DNA molecules. Increased solubility can be achieved by extending the PNA sequence with charged amino acid residues such as lysines [185] or by incorporating solubility enhancers (E-linkers) [186] prepared by replacing the nucleobases of the standard PNA monomers with either neutral or positively charged hydrophilic moieties. Though these E-linkers do not influence the hybridization performance of the PNAs, PNA probes with solubility enhancers have been found to be almost incapable of penetrating cell walls of a number of Gram-positive bacteria in whole cell FISH analysis [187], so they should not be used indiscriminately. A different solution has been to grow target cells in 10% LB media to overcome solubility limitations of PNAs [188,189].

A number of different analogues have been produced introducing different non-natural nucleobases into the PNAs [185], and some of these analogues resulted in a high preference for RNA binding over DNA binding [190,191].

PNA oligos are usually very short, since increasing the length generally results in decreased solubility [186], and shorter PNAs are more likely to efficiently enter cells [27]. For antisense applications in bacteria, 9–12-mer PNAs with low self-complementarity and an average GC content are recommended [192]. A low GC content, especially in short PNAs, could result in low binding affinity, whereas a high GC content could cause problems with respect to synthesis and solubility [183].

**Figure 11:**
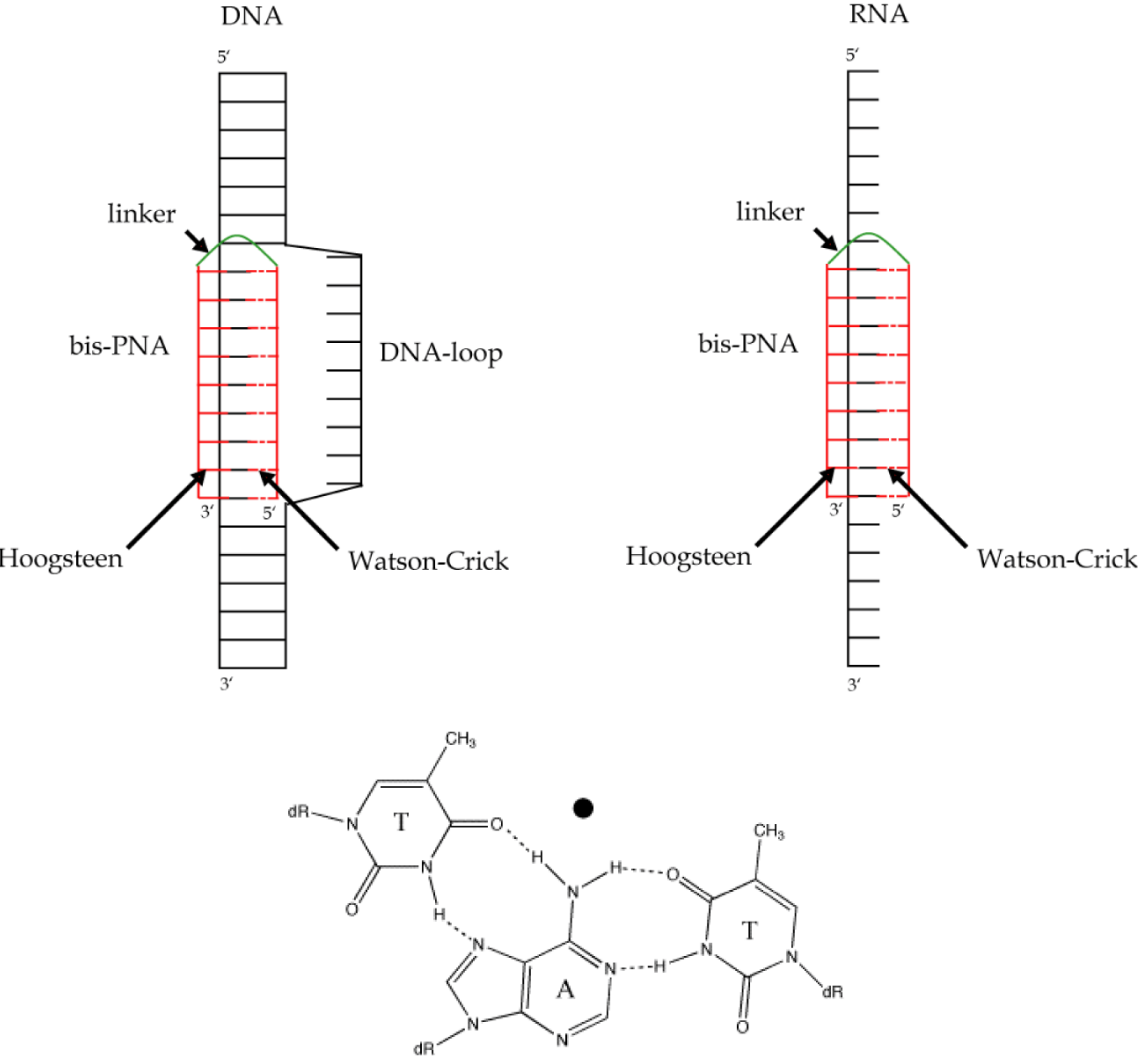
**Schematic representation of bis-PNA binding**. (Top) Bis-PNA (depicted in red) can bind to both DNA and RNA through Hoogsteen base pairing (unbroken line) and Watson-Crick base pairing (dotted line). (Bottom) An example on Hoogsteen (on the left) and Watson-Crick (on the right) base pairing is shown in detail.

Triplexes formed between one homopurine DNA or RNA strand (composed of adenine and guanine bases only) and two sequence-complementary PNA strands are extraordinarily stable [183]. One PNA strand binds through Watson-Crick base pairing (preferably in antiparallel orientation) and the other via Hoogsteen base pairing (preferably in parallel orientation). The PNA strands can be advantageously connected to each other covalently by a flexible linker (e.g. three 8-amino-3,6-dioxaoctanoic acid units) to create a bis-PNA (Fig. 11). Furthermore, the binding can be rendered pH independent by replacing cytosines in the Hoogsteen base pairing strand with pseudoisocytosines [193].

For Gram-negative bacteria, the peptidoglycan-layer, the lipid bilayer, and especially the external lipopolysaccharide (LPS) layer provide the bacteria with an efficient first-line of defence and the PNA with a major challenge [194]. PNAs are larger than most drugs (about 2000–4000 MW [195]) and are not readily taken up by bacteria, so different strategies have been developed to facilitate cellular uptake (see reviews by Nielsen [195-197]). Methods for physically disrupting cell membranes are limited to cells in culture, but strategies involving cationic liposomes and conjugation to fatty acids or peptides can be applied in tissues as well. Conjugation of PNAs to small cationic peptides termed cell-penetrating peptides (CPPs) appears to be the most efficient strategy for PNA cell entry [198]. (KFF)_3_K, shown by Vaara and Porro to have a cell wall-permeabilizing effect, has become one of the most widely used CPPs [199].

### 6.4 Applications of Peptide Nucleic Acids

PNA technology has been used to target a number of different *E. coli *RNAs and genes encoding essential proteins. These targets include the peptidyl transferase center, the α-sarcin loop [189,192] and domain II of 23S rRNA [200], the P15 loop region of RNase P RNA [153], and mRNAs encoding β-galactosidase (*lacZ*), β-lactamase (*bla*) [188], and the acyl carrier protein Acp (*acpP*) [192,201] (Table 2). The obtained results are not directly comparable due to differences in growth medium, starting inoculum etc, but the following will comprise an overview of the development.

**Table 2:** Studies on PNAs in bacteria.

PNA	Target	Test system	Reference
Triplex-forming bis-PNA	α-sarcin loop + peptidyl transferase center of 23S rRNA	*E. coli in vitro *system + solid and liquid culture	[189]
15-mer PNA	*lacZ *+ *bla*	*E. coli *K-12 and AS19 *in vitro + in vivo*	[188]
9–12-mer PNAs and (KFF)_3_K-PNAs	α-sarcin loop + peptidyl transferase center of 23S rRNA + *acpP *+ *lacZ*	HeLa cells infected with noninvasive *E. coli*	[192]
10–12-mer PNAs and peptide-PNAs	*phoB *+ *fmhB *+ *gyrA *+ *hmrB *+ *gfp*	*S. aureus *liquid culture	[205]
10-mer (KFF)_3_K-PNA	*acpP*	Mouse infected with *E. coli *K-12 and SM101	[201]
12-mer peptide-PNA	*gfp *+ *inhA*	*M. smegmatis *liquid cultures	[206]
14-mer (KFF)_3_K-PNA	P15 loop of RNase P RNA	*E. coli in vitro *+ *in vivo*	[153]
Triplex-forming (KFF)_3_K-bis-PNA	Domain II of 23S rRNA	*E. coli in vitro *system + solid and liquid culture	[200]
15–16-mer (KFF)_3_K-PNAs	*gyrA *+ *ompA *+ *lacZ*	*K. pneumoniae *liquid culture + IMR90 cells infected with *K. pneumoniae*	[204]
10–12-mer (KFF)_3_K-PNAs	*acpP *+ *lacZ*	*E. coli *K-12 liquid culture	[202]

Bis-PNAs targeting the peptidyl transferase center, the α-sarcin loop [189], and domain II [200] of 23S rRNA were shown to inhibit translation in a cell-free translation/transcription system. Substantially lower concentrations were required for *in vitro *inhibition when applying two different anti-*bla *PNAs and an anti-*lacZ *PNA [188]. Specific inhibition of the RNase P holoenzyme was also observed at very low concentrations when using a peptide-PNA targeting the P15 loop region of the RNase P RNA. It was shown to bind its target essentially irreversibly *in vitro *and disrupt local secondary structure in the catalytic core [153].

A rather moderate inhibition of cell growth in culture is a common feature of most of these PNAs, even though they are efficient inhibitors in cell-free systems. This is true at least for wildtype *E. coli *strains such as K-12 or DH5α. However, the PNAs have a greatly increased inhibitory effect on permeable mutant strains such as AS19 [153,188,189] and SM101 [201], and inefficient uptake and passage through the outer membrane is therefore considered to be the reason for this somewhat moderate cell growth inhibition observed in wildtype. Attachment of the CPP, (KFF)_3_K, to PNAs have been shown to improve the inhibitory effect. For instance, the aforementioned anti-α-sarcin loop bis-PNA was in a following study conjugated to (KFF)_3_K, and this resulting peptide-PNA exhibited a low minimal inhibitory concentration (MIC) for *E. coli *K-12. (KFF)_3_K was also coupled to an anti-*acpP *PNA. Here, the inhibitory effect was even greater. In fact, it was recently demonstrated that this peptide-PNA has a lower MIC than the conventional antibiotics ampicillin, chloramphenicol, streptomycin and trimethoprim and is more potent on a molar basis [202]. This study also indicated that peptide-PNAs accumulate in bacteria through rapid uptake and slow passive efflux, and that they mediate a long post-antibiotic effect of more than 11 hours. So even though cell entry is still a challenge, once inside the cells the peptide-PNAs appear to be retained for hours, unlike conventional antibiotics that efflux within minutes [203].

The application of PNAs has been taken a step further. To set up a simple model for the growth of an extracellular pathogen in a host, HeLa cell cultures were infected with non-invasive wildtype *E. coli*. The anti-*acpP *peptide-PNA was added immediately post-infection. The PNA was bactericidal at low micromolar concentrations, and no effect was visible for the HeLa cell growth. However, peptide-PNA is still, in animal models, inferior to conventional antibiotics in potency [192].

Another group tested this anti-*acpP *peptide-PNA in an animal disease model [201]. BALB/c mice were challenged by i.p. injection of a 90% lethal dose (LD_90_) of *E. coli *K-12 or LD_70 _of SM101 mutant strain with a defective outer membrane. Administering anti-*acpP *peptide-PNA intravenously to the mice rescued up to 60% and 100% of the infected animals, respectively.

The inhibitory effect of peptide-PNAs has recently also been demonstrated for another Gram-negative bacterium, the human pathogen *Klebsiella pneumoniae *[204]. Targeting essential genes *gyrA *(DNA gyrase subunit A) and *ompA *(major outer membrane protein A) resulted in inhibition of protein synthesis and growth but at rather high MICs. Furthermore, the levels of the two transcripts were strongly reduced indicating an antigene effect. This is possible, since PNAs can invade double-stranded DNA and form highly stable triplex-invasion complexes that are strong enough to inhibit gene transcription and regulation. Furthermore, human epithelial fibroblasts (IMR90) were infected with multiresistant *K. pneumoniae*, and peptide-PNA treatment was bactericidal without visible affect on IMR90 cell growth.

Though Gram-positive bacteria are important human pathogens, they have been given little attention as target candidates for PNAs. Nekhotiaeva *et al*. recently demonstrated that peptide-PNAs are indeed effective in inhibiting growth of Gram-positive bacterium *S. aureus *[205]. (KFF)_3_K-PNAs were used to target chromosomally encoded alkaline phosphatase (*phoB*), putative peptidoglycan pentaglycine interpeptide biosynthesis protein (*fmhB*), gyrase A (*gyrA*) and HmrB protein (*hrmB*, which is an ortholog of the *E. coli acpP *gene). Generally, at least 10 μM concentrations were required for inhibition.

Passage across the outer membrane of Gram-negative bacteria has been indicated to be the rate-limiting step of peptide-PNA uptake [198]. Gram-positive bacteria should in their lack of an outer membrane pose an easier target, but it appears that CPPs are still required for cellular uptake. The mechanism has not yet been elucidated, but 'direct' cell permeation has been suggested as the most likely explanation for peptide-PNA entry into both *S. aureus *and *E. coli *[198,205].

As mentioned in section 6.2, the outer lipid bilayer of mycobacteria has an exceptionally low permeability. Nevertheless, peptide-PNAs have been shown to enter mycobacterial cells and inhibit growth by antisense inhibition [206]. (KFF)_3_K-PNAs were used to target *inhA*, which encodes the essential protein enoyl reductase, and *M. smegmatis *growth was inhibited at fairly low MICs.

Further optimization of peptide-PNAs to increase efficiency is still required. The linker connecting peptide and PNA is one target of optimization [153], since Good *et al*. [192] showed the linker not to be a 'silent player'.

The cell delivery efficiency of CPPs is another target for optimization. The widely used (KFF)_3_K carrier peptide has been found to induce haemolysis in human erythrocytes [199] and histamine release in some mammals [205], so alternative CPPs would be needed for broad medical applications of PNAs. New peptides have been proven efficient in PNA delivery into *S. aureus *[205] as well as into *M. smegmatis *[206]. Thus, improvement of carrier peptides should be possible, though these new CPPs still need further characterization.

PNA targeting has not been limited to rRNA and mRNA, also non-coding regulatory RNAs have been used as targets for PNAs [207]. Sok-antisense-RNA inhibits synthesis of Hok protein, which induces host cell killing. Introducing (KFF)_3_K-PNAs against plasmid-encoded Sok-antisense-RNA leads to Hok protein synthesis and cell killing in *E. coli *(for description of *hok*/*sok *toxin-antitoxin system see [208]), and the (KFF)_3_K-PNA was actually more inhibitory than rifampicin. However, it still remains to be demonstrated that (KFF)_3_K-PNA targeting antitoxins can activate suicide in plasmid-free *E. coli *cells.

### 6.5 Phosphorodiamidate Morpholino Oligomers

Phosphorodiamidate morpholino oligomers (PMOs, Fig. 9) are oligonucleotide mimics, in which the deoxyriboses of DNA have been replaced by morpholine rings coupled by non-ionic phosphorodiamidate intersubunit linkages [209,210]. Despite being uncharged, the PMOs show excellent aqueous solubility, possibly due to good stacking of the bases and shielding of hydrophobic faces [209]. Furthermore, PMOs have displayed resistance to a variety of nucleases, proteases, esterases, and degradative enzymes in serum and liver homogenates [211], and like PNAs their mechanism of action is RNase H-independent and involves sequence-specific binding and steric blocking of ribosomal assembly and hence translation (Fig. 1). However, the binding affinity of PMOs is lower than for the equivalent PNAs [212].

### 6.6 Applications of Phosphorodiamidate Morpholino Oligomers

PMO-mediated translation inhibition in bacteria has mainly been focused on targeting the mRNA encoding the essential acyl carrier protein Acp (*acpP*) of *E. coli *(Table 3), though the effects of PMOs on *Bacillus anthracis *are currently being studied [213]. As for PNAs, the outer membrane of the Gram-negative *E. coli *poses a barrier for PMO cell uptake, and 10–20-mer PMOs were only shown to be effective in mutants with a defective outer membrane [212,214]. A specific 11-mer anti-*acpP *PMO did, however, briefly inhibit growth of wild-type *E. coli *[215].

**Table 3:** Studies on PMOs in bacteria.

PMO	Target	Test system	Reference
20–21-mer PMOs, (KFF)_3_KC-PMOs and rTat-PMOs	16S rRNA + *acpP *+ *lacI*	*E. coli *BL21(DE3), SM101 and SM105 in solid + liquid culture	[214]
7–20-mer PMO	*acpP*	*E. coli *SM101 and AS19 and HeLa liquid culture	[212]
11-mer PMO	*acpP*	*E. coli *SM105 and AS19 in liquid culture + in mice	[215]
10–11-mer PMOs with/without 4 different CPPs	*acpP*	*E. coli *K-12 and E2348/69 (EPEC) and *S. enterica *ser. Typh. liquid culture + EPEC in Caco-2 cells	[216]
11-mer (RFF)_3_RXB-PMO	*acpP*	Mouse infected with *E. coli *K-12	[217]

To improve PMO efficiency, conjugation to CPPs was tested. (KFF)_3_KC, resembling the (KFF)_3_K peptide used to promote cell uptake of PNAs (see section 6.2), was effective in facilitating cell uptake of 20-mer PMOs [214]. The free peptide was shown to be toxic at 20 μM, but conjugation to the PMO apparently eliminated this toxicity.

Using the 11-mer anti-*acpP *PMO, another three CPPs were shown to efficiently assist in transporting the PMO into cells [216]. In this study, pathogenic strains of *E. coli *(enteropathogenic *E. coli *[EPEC]) and *S. enterica *serovar Typhimurium appeared more susceptible to peptide-PMO inhibition than wildtype *E. coli*, possibly due to different extents of peptide hydrolysis in the different strains. EPEC growth in tissue culture could also be inhibited by the peptide-PMOs, but their efficiency in reducing bacterial viability was orders of magnitude greater in tissue culture than in liquid culture. This may be due to differences in bacterial growth in the two types of culture.

The inhibitory effect, though modest, of the anti-*acpP *PMO in a mouse model of *E. coli *peritonitis was the first demonstration of antisense DNA analogues inhibiting bacterial growth in an animal model [215]. The conjugate of this PMO and (RFF)_3_RXB-peptide was shown to be about 50–100 times more potent than the single PMO in the mouse model [217]. In the mouse model of *E. coli *peritonitis, the anti-*acpP *peptide-PMO was found to reduce the number of colony-forming units (CFU) in the blood and promote survival at a potency at least 15 times greater than ampicillin. Though the results suggested high doses of the conjugate to be toxic, preliminary toxicology data on treatment with about 20 times higher doses of anti-*acpP *peptide-PMO showed no apparent toxicity [217]. Since the possible toxicity is thought to be caused by the peptide moiety and not the PMO (PMO compounds are considered safe in humans [218]), further optimizations on this part of the compound will be necessary, unless the toxicological studies can acquit this peptide of toxicity.

The therapeutic use of antisense PMOs seem highly possible, and further pharmokinetic and toxicological studies will help improve this compound for antibacterial treatment.

## 7. Concluding remarks

The apparent and alluring simplicity of antisense sequence inhibition of bacterial growth has turned out to be simply apparent, and the antisense approach now appears far more complicated and challenging than first thought. Parameters such as degradation resistance, binding efficiency, solubility, intracellular concentration and cellular delivery must be considered carefully, and further optimization on these parameters is still required despite the emergence of the first antisense-based drug almost a decade ago. Pharmaceutical formulations of antisense compounds with the same potency as antibiotics have not yet been developed, though they become increasingly more needed as bacterial resistance spread. The substantially modified PNAs and PMOs show promising results and may in time become the new medical weapons against the elusive pathogenic bacteria. In any case, we can not afford not to continue this research.

## Abbreviations

2'-OMe 2'-*O*-methylribonucleotide

asRNA antisense RNA

cat chloramphenicol acetyltransferase

CFU colony-forming units

CPP cell-penetrating peptide

dsRNA double-stranded RNA

EGS external guide sequence

EPEC enteropathogenic *E. coli*

FDA The Food and Drug Administration

HH hammerhead

i.p. intraperitoneal

IC_50 _concentration required for 50% inhibition

LD_70/90 _70%/90% lethal dose

LNA locked nucleic acids

MALDI-TOF matrix-assisted laser desorption/ionization time of flight

MIC minimal inhibitory concentration

MoMuLV moloney murine leukemia virus

MRSA methicillin-resistant *S. aureus*

ODN oligodeoxynucleotide

PBP2a penicillin-binding protein 2a

PBS primer binding site

PMO phosphorodiamidate morpholino oligomer

PNA peptide nucleic acid

PS-FANA phosphorothioate-linked 2'-deoxy-2'-fluoro-D-arabinonucleic acid

PS-ODN phosphorothioate oligodeoxynucleotide

ptRNA precursor tRNA

RBS ribosome binding region

RNAi RNA interference

rRNA ribosomal RNA

ssDNA single-stranded DNA

## Competing interests

The author(s) declare that they have no competing interests.

## Authors' contributions

LCVR formulated the content, performed the literature search, drafted the manuscript and created the illustrations. KKM contributed to revising the manuscript and created the illustration of chemical structures. HUSP contributed to revising the manuscript. All authors read and approved the final manuscript.
